# Development of multiphasic CT-based delta-radiomics model for predicting postoperative recurrence risk in bladder cancer

**DOI:** 10.1186/s12880-026-02554-2

**Published:** 2026-07-07

**Authors:** Weiwei Liu, Bingxin Gong, Guilin Zhang, Yusheng Guo, Xiaona Fu, Jie Lou, Peng Sun, Yi Li, Shanshan Jiang, Chao Huang, Lian Yang

**Affiliations:** 1 Department of Radiology, Union Hospital, Tongji Medical College, Huazhong University of Science and Technology, Jiefang Avenue #1277, Wuhan, 430022 China; 2 Hubei Provincial Clinical Research Center for Precision Radiology & Interventional Medicine, Wuhan, 430022 China; 3 Hubei Key Laboratory of Molecular Imaging, Wuhan, 430022 China; 4 Institute of Research and Clinical Innovations, Neusoft Medical Systems Co. Ltd, Shenyang, 110167 China; 5 Department of Urology, Tongji Medical College, Union Hospital, Huazhong University of Science and Technology, Wuhan, 430022 China

**Keywords:** Delta radiomics, Bladder cancer, Recurrence, Machine learning, Immune-related pathways

## Abstract

**Background:**

This study aimed to develop a CT-based delta-radiomics model for personalized prediction of postoperative prognosis in bladder cancer patients and identification of differentially expressed genes associated with tumour recurrence.

**Materials and methods:**

This retrospective study included 316 patients with bladder cancer from Wuhan Union Hospital who underwent preoperative unenhanced and arterial-phase CT before surgical resection. Patients were randomly divided into training and internal validation cohorts at an 8:2 ratio. Radiomic features were extracted from tumor volumes of interest on both CT phases, and delta-radiomic features were calculated as arterial-phase features minus unenhanced-phase features. Feature selection was performed within the training cohort using statistical filtering, correlation analysis, and LASSO regression. Multiple machine-learning classifiers were evaluated, and a combined clinico-radiomic model was constructed by integrating the delta-radiomics score with selected clinical predictors. Model performance was assessed using ROC analysis, calibration, decision curve analysis, DeLong testing, and repeated stratified random splitting. An additional TCIA cohort of 33 patients was used for exploratory transcriptomic analysis.

**Results:**

After feature selection, nine delta-radiomic features derived from paired unenhanced and arterial-phase CT images were retained for model construction. The LightGBM-based delta-radiomics model achieved AUCs of 0.837 and 0.822 in the training and validation cohorts, respectively. The combined clinical-radiomics model, constructed by integrating the delta-radiomics Rad-score with muscle invasion as a clinical predictor, achieved the best discrimination, with AUCs of 0.860 and 0.861 in the training and validation cohorts, respectively, and showed stable validation performance in 100 repeated splits. High radiomics scores were associated with significantly shorter recurrence-free survival. Exploratory transcriptomic analysis showed enrichment of immune-related pathways in the low-risk radiomic subgroup.

**Conclusions:**

Multiphasic CT-based delta-radiomics provides a non-invasive approach for predicting postoperative recurrence and stratifying prognosis in bladder cancer. A combined clinico-radiomic model incorporating delta-radiomics and muscle invasion achieved superior predictive performance and may support individualized postoperative risk assessment.

**Supplementary Information:**

The online version contains supplementary material available at https://doi.org/10.1186/s12880-026-02554-2.

## Highlights

This study is the first to develop a novel delta-radiomics framework leveraging unenhanced and arterial-phase CT scans to predict postoperative recurrence in bladder cancer, outperforming single-phase radiomics (validation AUC: 0.822 vs. 0.783).

Combined delta-radiomics signatures with clinical risk factors using LightGBM, achieving superior recurrence prediction (training AUC: 0.860) and enabling significant risk stratification for recurrence-free survival (validation HR = 3.58, *p* < 0.001).

Identified enrichment of immune-related genes in low radiomic risk patients via RNA sequencing, revealing biological mechanisms underlying recurrence susceptibility.

**Supplementary Information:**

The online version contains supplementary material available at https://doi.org/10.1186/s12880-026-02554-2.

## Introduction

Bladder cancer (BLCA) is the tenth most prevalent malignancy and the second most common urological cancer worldwide [1]. Histologically, it can be classified into non-muscle-invasive bladder cancer (NMIBC) and muscle-invasive bladder cancer (MIBC). Approximately 75% of patients are NMIBC at initial diagnosis [2] and are primarily treated by transurethral resection of bladder tumours (TURBT), but the recurrence rate is as high as 78%, with a 45% risk of progression within 5 years [3]. Radical cystectomy remains the standard treatment for MIBC. However, approximately 50% of patients experience postoperative recurrence or metastasis, often leading to death within 2 years [4, 5]. While adjuvant therapies (e.g., chemotherapy, radiotherapy) are used for high-risk cases based on pathology, the overall prognosis for MIBC patients remains poor [6]. In BLCA, the current prognostic stratification relies mainly on the TNM staging system [7, 8], but significant survival differences exist among patients with the same stage. Therefore, the development of accurate prognostic risk stratification tools for BLCA is clinically important to guide individualised treatment decisions and optimise follow-up strategies.

Computed tomography (CT), as an important tool for the diagnosis and staging of bladder cancer, can avoid invasive tests such as cystoscopy when clearly displaying bladder tumours [9]. Radiomics, as an emerging method of image analysis, provides key information for clinical decision support systems by extracting quantitative features in a high-throughput manner, thus enhancing the accuracy of diagnosis, prognosis and prediction [10, 11]. The method can transform medical images into multidimensional feature sets that reveal tumour pathophysiology, which has significant application potential. However, how to efficiently screen, validate and utilise these massive features remains a core challenge in current radiomics research. Machine learning algorithms have effectively solved the high-dimensional feature processing challenge by optimising feature selection and combination strategies [12–14]. They are able to deeply explore the association between radiomics features and tumour biological characteristics (e.g. heterogeneity, treatment sensitivity), and thus achieve accurate prediction of disease progression and patient prognosis. Currently, machine learning-based radiomics models have been successfully applied to precise staging, immune microenvironment assessment and treatment response prediction in a variety of malignant tumours, such as oesophageal cancer, breast cancer and lung cancer [12–16].

Delta radiomics captures the evolution of tumour biological behaviour by quantifying the dynamic net change of histological features in multi-phase CT imaging [17, 18]. In particular, arterial-phase imaging enhances the display of tumour angiogenesis (an invasive phenotypic marker), whereas the unenhance-phase provides basic anatomical information. By integrating the differences in dual-phase features, delta radiomics could theoretically reveal pathophysiological processes (e.g., angiogenesis or intratumoural hypoxia) associated with recurrence. Previous studies have confirmed that delta features significantly correlate with tumour treatment response and prognosis [19, 20], and that the fusion of multi-phase features in hepatocellular and pancreatic cancers improves diagnostic accuracy [13]. However, there is a lack of systematic validation of whether delta radiomics models based on plain versus arterial CT are superior to single-phase phase models for bladder cancer recurrence prediction. Meanwhile, the incremental predictive value of delta features combined with clinical variables to construct multimodal models remains to be explicitly quantified.

This study aims to develop an integrated machine learning model that combines clinical parameters with radiomic features, specifically leveraging delta radiomics derived from both arterial phase and unenhanced CT scans, for predicting recurrence risk and long-term clinical outcomes in BLCA patients. Furthermore, we assessed the incremental prognostic value contributed by radiomics beyond established evidence-based clinical and radiological factors. In parallel, utilizing the The Cancer Image Archive (TCIA)-BLCA cohort, we identified recurrence-associated differentially expressed genes to elucidate their underlying biological functions.

## Materials and methods

This study was conducted in accordance with the principles of the Declaration of Helsinki. Ethical approval, including a waiver of informed consent, was granted by the Institutional Review Board of Tongji Medical College, Huazhong University of Science and Technology for this retrospective cohort analysis. All clinical data were anonymized prior to retrospective analysis by the research team.

### Study sample

**Fig. 1:**
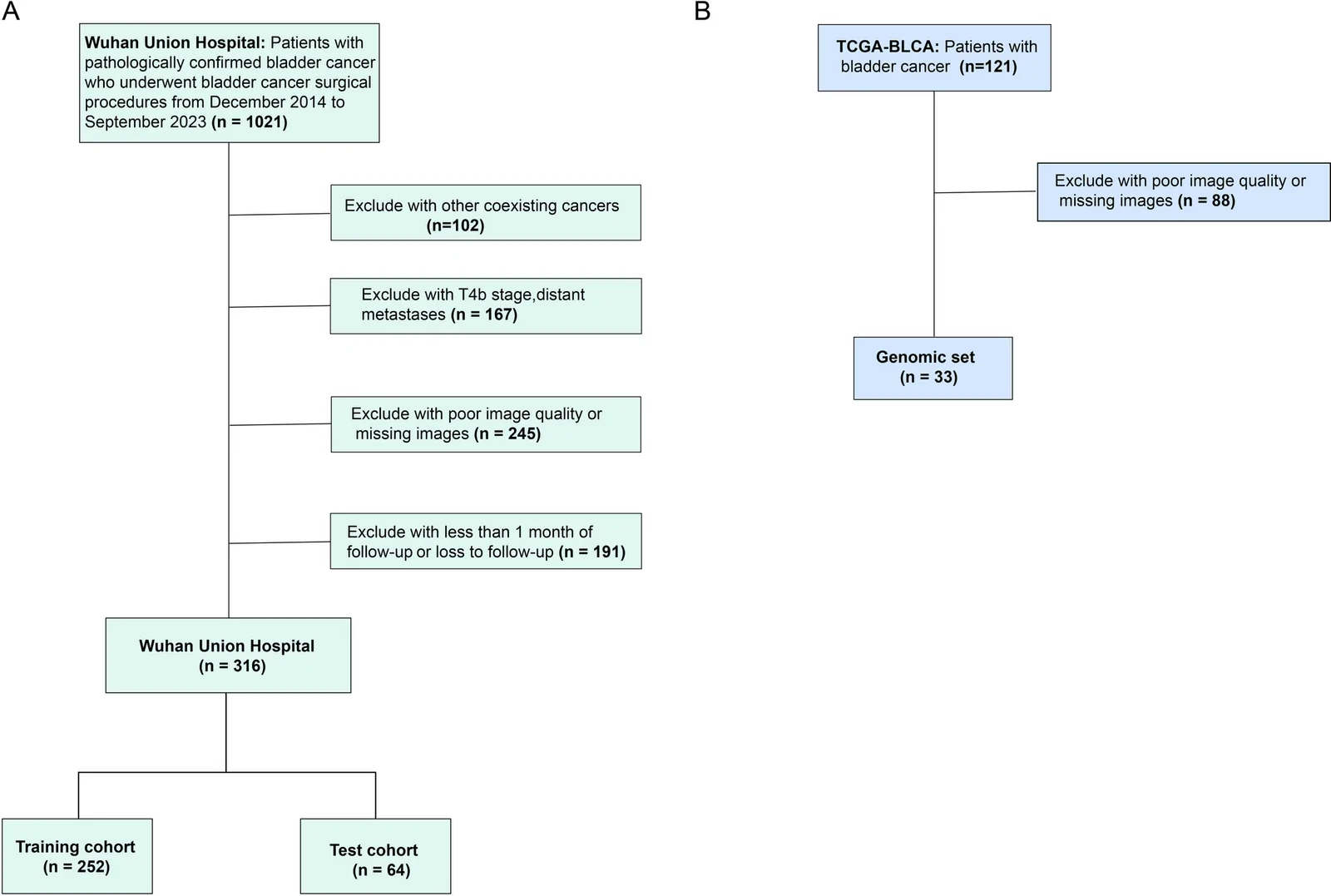
Flowchart of the study datasets: (**A**) training and validation cohorts to predict RFS in patients with bladder cancer, and (**B**) genomics set. TCGA-BLCA datasets are publicly available and were downloaded from the cancer imaging archive and the cancer Genome Atlas. RFS, recurrence-free survival

This retrospective study included 316 BLCA patients from Wuhan Union Hospital (December 2014 - September 2023) and 33 BLCA patients from TCIA who underwent bladder cancer resection (Fig. 1A, B). The inclusion criteria for patients from Wuhan Union Medical College Hospital were as follows: (a) pathological diagnosis of bladder cancer, (b) available clinical information, (c) age > 18 years and (d) patients underwent non-contrast and enhanced abdominal CT examination within 4 weeks before cystectomy for bladder cancer. Exclusion criteria were as follows: (a) other malignancies in combination, (b) less than one month of follow-up or lack of follow-up, (c) patients with T4b stage, distant metastases (M1 stage) and (d) image artefacts or poor quality of image alignment.

For model training, patients at the Wuhan centre were randomly assigned to the training and testing groups in a ratio of 8:2.

### CT study protocols

CT images are obtained in cross-sectional view, including non-contrast CT and arterial phase CT of the bladder, imaging protocols are detailed in Table S1.

### Clinical characteristics and outcomes

Patient demographic and pathological characteristics were recorded preoperatively. Demographic data encompassed age, sex, body mass index (BMI), albumin to globulin ratio (AGR), neutrophil-to-lymphocyte ratio (NLR), platelet-to-lymphocyte ratio (PLR), smoking and alcohol consumption history, and comorbidities including hypertension, diabetes mellitus, and cardiovascular disease, along with prior chemotherapy exposure. Pathological parameters, assessed within four weeks preceding CT imaging, comprised pathologic tumor grade, pathologic stage, and whether muscle invasion was present.

Within this study, recurrence was defined as either disease relapse or death from any cause. The primary endpoints were recurrence-free survival (RFS) and overall survival (OS). RFS is the time from the end of treatment to the first tumour recurrence or death from any cause after the patient has been treated surgically to achieve complete remission (CR). OS was defined as the duration from the initial surgical intervention until death due to any cause.

### CT images acquisition and segmentation

Bladder cancer imaging Segmentation data were prepared following the nnUNetv2 framework architecture (publicly accessible at: https://github.com/MIC-DKFZ/nnUNet/blob/master/documentation/how_to_use_nnunet.md). This pipeline performed automated preprocessing—including intensity normalization and resampling—while extracting key data attributes (e.g., voxel dimensions, spatial resolution, intensity distribution). These analyses subsequently generated optimized configurations for both 2D U-Net and 3D full-resolution U-Net architectures. Five-fold cross-validation was employed to train models using both configuration types. All ROIs were reviewed and manually refined using ITK-SNAP by a board-certified radiologist (G.B.X., > 5 years of experience). To assess interobserver segmentation reproducibility, 50 randomly selected cases were independently refined by a second board-certified radiologist (G.L.Z., > 5 years of experience) blinded to clinical information and recurrence outcomes. Agreement between the two refined VOI sets was evaluated using the Dice similarity coefficient and the intraclass correlation coefficient (ICC) for segmentation-derived measurements.

### Radiomics feature extraction

Radiomic features were extracted from each volume of interest (VOI) using the FAE software package (version 0.5.2; https://github.com/salan668/FAE), an open-source platform built upon PyRadiomics [21]. Feature extraction strictly followed the guidelines established by the Image Biomarker Standardization Initiative (IBSI) [22], performed independently on unenhanced and arterial phase CT images. Extracted features encompassed shape descriptors, first-order statistics, textural patterns, and wavelet transforms. Ultimately, a total of 3668 radiomic features were derived per patient from the segmented VOIs on CT imaging. Before outcome-related modeling, the dataset was randomly split into training and internal validation cohorts at an 8:2 ratio. All preprocessing, feature selection, tuning, model fitting, and cutoff determination were performed within the training cohort, and robustness was assessed by repeating recurrence-stratified 8:2 splitting and the full training-only workflow 100 times.

### Feature selection

Feature selection was performed only after the training-validation split and exclusively within the training cohort to avoid information leakage. Z-score normalization parameters were estimated from the training cohort and then applied unchanged to the validation cohort. Within the training cohort, recurrence-associated features were first screened using independent-samples t-tests (*p* < 0.05), followed by removal of highly correlated features using Pearson correlation analysis (|r| > 0.9). LASSO regression with five-fold cross-validation was then used to determine the optimal penalty coefficient and retain features with non-zero coefficients. The final selected feature set was locked before being applied to the held-out validation cohort.

Radiomics features of the tumour region were independently extracted from unenhanced (unenhanced-RFs) and arterial-phase CT (arterial-RFs).

Delta-radiomics features (delta-RFs) were derived by calculating the arithmetic difference between radiomics features extracted from arterial phase CT and those from unenhanced phase CT. This relationship is expressed as:

$$\rm RF_{arterial}-RF_{unenhanced} = RF_{delta}$$

### Radiomics features selection and clinical-radiomics model construction

**Fig. 2:**
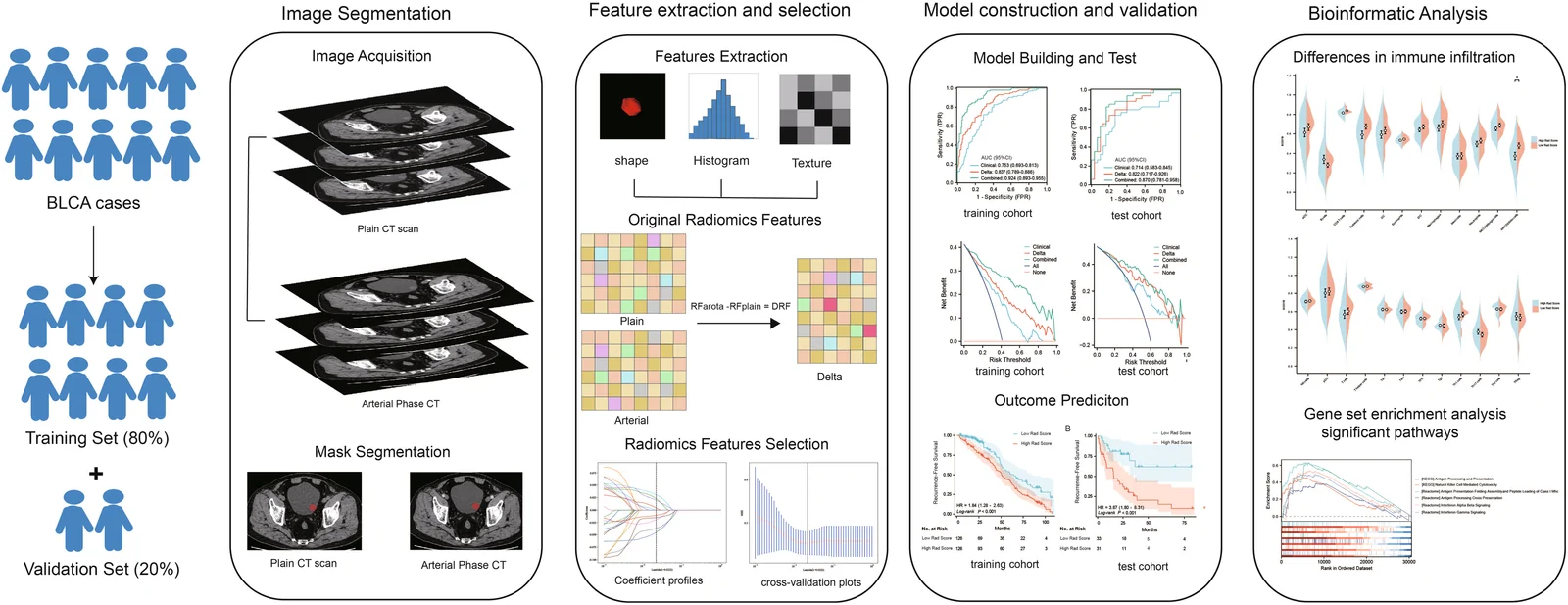
Workflow of the clinical-radiomics model development

Eight supervised classifiers, including logistic regression, Extra Trees, k-nearest neighbors, support vector machine, random forest, LightGBM, multilayer perceptron, and XGBoost, were evaluated to develop radiomics models using the selected radiomics features. Hyperparameters were optimized by grid search within the training cohort, and the best-performing model was selected for subsequent analyses. The probabilistic output of the final radiomics model was defined as the Rad-score. Binary recurrence status was used as the endpoint, with delta-RFs as predictors. To assess the incremental value of delta-RFs, models based on unenhanced-RFs or arterial-RFs alone were constructed using the same workflow. Figure 2 illustrates the complete radiomic model development workflow.

Candidate clinical variables were selected according to clinical relevance and data availability. Univariable logistic regression was performed in the training cohort, and variables with *P* < 0.05 or clear clinical relevance were entered into multivariable logistic regression. Variables retained in the final model were used to build the clinical model.

To develop the clinical-radiomics model, the delta-radiomics score was integrated with selected clinical predictors, and seven candidate algorithms were evaluated. Model performance was assessed using AUC-ROC, calibration curves, and decision curve analysis (DCA) [23], with ROC comparisons performed using DeLong’s test [24]. For all models, the optimal cutoff was determined in the training cohort by maximizing the Youden index and then applied unchanged to the validation cohort to calculate accuracy, sensitivity, specificity, positive predictive value, and negative predictive value.

### Outcome prediction and exploring biologic functions

Using median thresholds derived from the training cohort, patients were stratified into high- and low-risk groups based on the radiomics score (median = 0.57) and the combined model score (median = 0.66). Kaplan-Meier survival analysis with log-rank testing was used to compare overall survival (OS) and recurrence-free survival (RFS) across the different risk strata. Within the TCIA validation cohort (*n* = 33), patients were similarly dichotomized into low- and high-risk subgroups according to data completeness requirements for arterial-phase CT, unenhanced-phase CT, and RNA sequencing.

### Statistical analysis

Categorical variables underwent comparative analysis using χ² or Fisher’s exact tests, while continuous variables were evaluated via Student’s t-tests or Mann-Whitney U tests. Model discrimination performance was quantified through the ROC, accuracy, sensitivity, and specificity. All analytical workflows including feature selection and predictive modeling were executed in Python 3.8.5. Survival outcomes (OS and RFS) were estimated using Kaplan-Meier methodology, with between-strata differences assessed through log-rank testing. Statistical significance was defined as a two-tailed *P*-value < 0.05.

## Results

### Characteristics of the study sample

Table 1 details the clinicopathological characteristics of patients in the training cohort (*n* = 252) and internal validation cohort (*n* = 64), and there were no statistically significant differences between the two groups. All 316 enrolled patients underwent surgery for bladder cancer. The cohort comprised 260 males (82.3%), with a median age of 64 years. Non-muscle-invasive bladder cancer (NMIBC) was diagnosed in 227 patients (71.8%), and 183 individuals (57.9%) received adjuvant chemotherapy. Clinicopathological data for the TCIA cohort are presented in Table S2.

### Interobserver segmentation reproducibility

The interobserver analysis demonstrated good segmentation reproducibility, with a mean Dice similarity coefficient of [0.88, 95%CI:0.86–0.90] and an ICC of [0.94, 95% CI: 0.93–0.95] for segmentation-derived measurements (Table S3).

**Table 1:** Characteristics of patients in the training and internal validation cohorts

Characteristics	Training Set (*n* = 252)	Validation Set (*n* = 64)	*P* value
Patient characteristics			
Gender, n (%)			0.18
Male	211 (83.7%)	49 (76.6%)	
Female	41 (16.3%)	15 (23.4%)	
Pathologic tumor grade, n (%)			0.72
Low grade/ PUNLMP	114 (45.4%)	27 (42.9%)	
High grade	137 (54.6%)	36 (57.1%)	
Pathologic T stage, n (%)			0.42
< T2	178 (71.5%)	49 (76.6%)	
≥ T2	71 (28.5%)	15 (23.4%)	
BMI(kg/m^2^), mean (SD)	23.9 (3.1)	23.4 (3.7)	0.28
Age, mean (SD)	63.5 (12.8)	63 (10.8)	0.77
NLR, mean (SD)	3.9 (4.5)	4.4 (4.0)	0.42
PLR, mean (SD)	160.5 (126.3)	166.0 (96.3)	0.75
Adjuvant chemotherapy, n (%)			0.39
Yes	149 (59.1%)	34 (53.1%)	
No	103 (40.9%)	30 (46.9%)	
Hypertension, n (%)			0.76
Yes	70 (27.8%)	19 (29.7%)	
No	182 (72.2%)	45 (70.3%)	
Drinking, n (%)			0.25
Yes	56 (22.2%)	10 (15.6%)	
No	196 (77.8%)	54 (84.4%)	
Smoking, n (%)			0.06
Yes	82 (32.5%)	13 (20.3%)	
No	170 (67.5%)	51 (79.7%)	
AGR, mean (SD)	1.6 (0.3)	1.6 (0.4)	0.44
Cardiovascular disease, n (%)			0.42
Yes	23 (9.1%)	8 (12.5%)	
No	229 (90.9%)	56 (87.5%)	
Diabetes, n (%)			0.96
Yes	36 (14.3%)	9 (14.1%)	
No	216 (85.7%)	55 (85.9%)	

Abbreviations: SD, standard deviation; NLR, neutrophil-to-lymphocyte ratio; PLR, platelet-to-lymphocyte ratio; AGR, albumin to globulin ratio; BMI, body mass index; PUNLMP, papillary urothelial neoplasms of low malignant potential

### Feature extraction and feature dimensionality reduction

A total of 3,668 radiomic features were extracted from unenhanced and arterial-phase CT images, from which delta-radiomic features were calculated. Feature selection was performed using independent-samples t-tests, Pearson correlation filtering (|ρ| > 0.9), and LASSO regression with 5-fold cross-validation. The LASSO-based feature selection procedures for the unenhanced, arterial-phase, and delta-radiomics models are illustrated in Figure S1, including coefficient profiles, cross-validation curves, and feature-weight plots. Nine delta-radiomic features were retained for the final model at the optimal penalty coefficient of α = 0.022. The detailed information and importance scores of the selected features are provided in Table S4 and Figure S2, respectively.

### Construction of radiomics model

Radiomic features were extracted from unenhanced and arterial-phase pre-cystectomy CT images, and delta radiomic features were further generated to capture inter-phase radiomic changes. To identify the optimal modeling strategy, the selected features were fitted using several machine-learning classifiers. The detailed hyperparameter configurations of the eight machine-learning classifiers are provided in Table S5.

Among evaluated classifiers, LightGBM demonstrated superior predictive performance for the delta radiomics model, achieving a training cohort AUC of 0.84 (95% CI: 0.789–0.886) with accuracy, sensitivity, specificity and F1 score of 0.74, 0.84, 0.68 and 0.75 respectively. Nevertheless, the other seven alternative machine learning classifiers maintained competent predictive accuracy (> 0.7 AUC for MLP, LightGBM, and XGBoost). Complete predictive metrics for all eight classifiers are detailed in Table S6. Validation performance remained robust (AUC 0.82, 95% CI: 0.717–0.926) (Figure S3A, B). Based on the DeLong test, the integrated discrimination improvement (IDI) ranged from − 0.020 to 0.149 in the training cohort and from 0.000 to 0.046 in the validation cohort, with several comparisons achieving statistical significance (Table S7). Comparative analysis revealed delta features significantly outperformed both unenhanced and arterial radiomics features across training and validation datasets, the delta model exhibited optimal calibration performance across both the training and validation cohorts (Figure S3C, D). DCA indicated that the integrated probability (Inte-prob) yielded superior net clinical benefit compared to the radiomics-derived probability (Rad-prob) (Figure S3E, F). Figure S3G-J presents the confusion matrices quantifying the models’ performance in recurrence prediction.

### Construction of clinical model

**Fig. 3:**
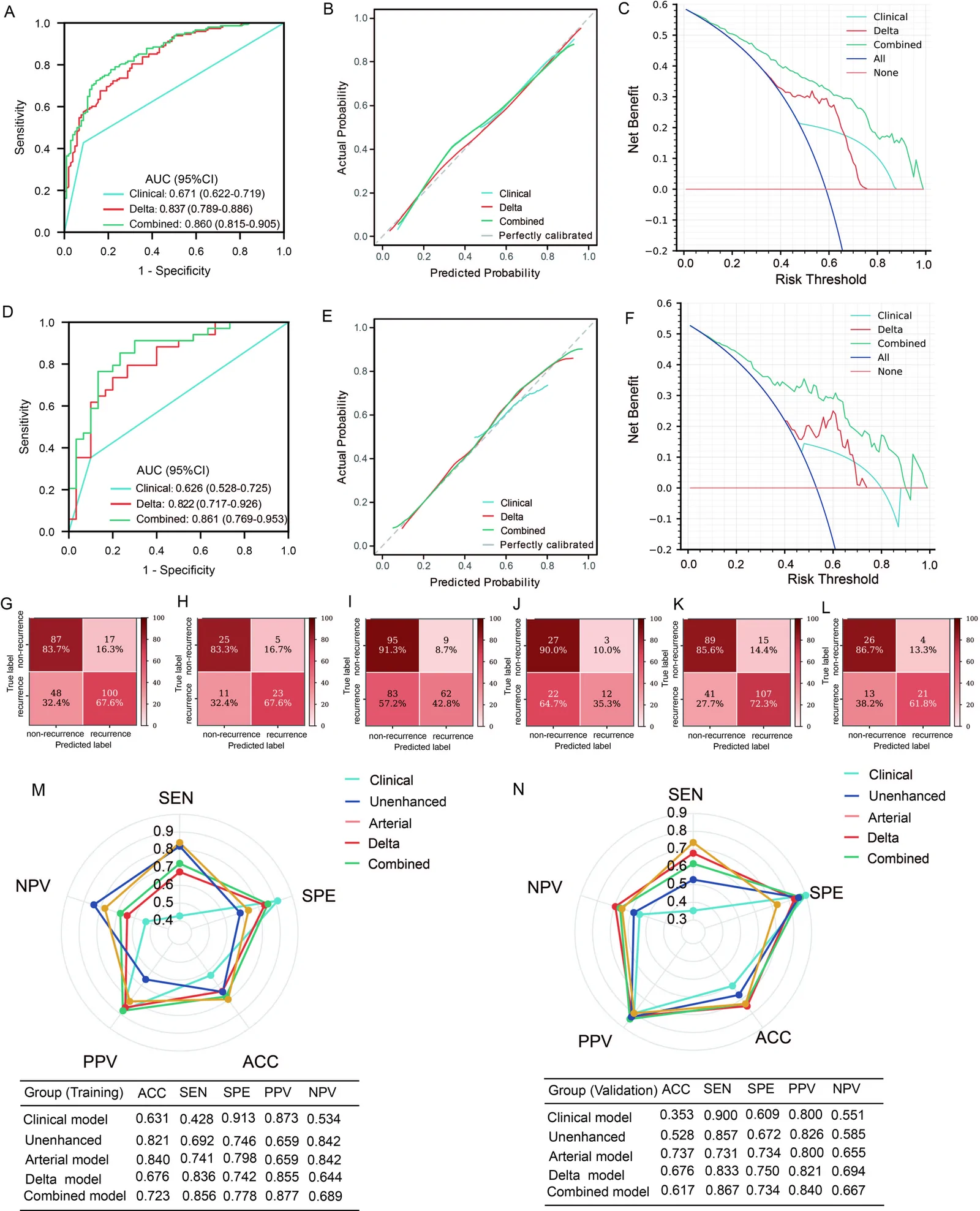
Comparative performance assessment of three models. Receiver operating characteristic (ROC) curves: Discrimination performance of Clinical, Delta, and Combined models in (**A**) training and (**D**) validation cohorts. Calibration plots: Agreement between predicted probabilities and observed outcomes for (**B**) training and (**E**) validation sets. Decision curve analysis (DCA): Clinical net benefit across threshold probabilities in (**C**) training and (**F**) validation cohorts. Confusion matrices: Classification performance of Delta training and validation (**G**,** H**), Clinical training and validation (**I**,** J**), Combined training and validation (**K**,** L**). Radar plots comparison metrics of all the models on the training (**M**) and validation cohorts (**N**). ACC, accuracy; SEN, sensitivity; SPE, specificity; PPV, positive predictive value; NPV, negative predictive value

In the clinical analysis, candidate clinical variables were evaluated using univariable and multivariable logistic regression in the training cohort. Pathologic T stage was the only variable that remained significant in the multivariable logistic regression analysis (OR = 7.15, 95% CI: 3.07–16.65, *P* < 0.001) (Table S8). Accordingly, the final clinical model was constructed using logistic regression with muscle invasion as the sole clinical predictor., achieving an AUC of 0.671 (95% CI: 0.622–0.719) in the training cohort with corresponding precision, sensitivity, and specificity of 0.63, 0.43, and 0.91. Validation in the test cohort yielded an AUC of 0.626 (95% CI: 0.528–0.725), with precision, sensitivity, and specificity values of 0.35, 0.90, and 0.61 respectively (Fig. 3A, D).

### Development of combined model

The combined clinico-radiomic model integrated the delta-radiomics score and muscle invasion. Seven machine-learning classifiers were compared using ROC, calibration, and decision curve analyses in the training and validation cohorts (Figure S4). Logistic regression showed stable discrimination, calibration, and clinical net benefit across cohorts and was therefore selected as the final algorithm. The final selected hyperparameters of the seven machine-learning classifiers evaluated for the combined clinico-radiomic model are provided in Table S9. All three models (clinical, delta-radiomics, and combined) demonstrated concordant predictive trends where elevated scores correlated with increased recurrence risk (Figure S5). The combined model exhibited superior discriminative performance in both training (AUC 0.860, 95% CI: 0.815–0.905) and validation cohorts (AUC 0.861, 95% CI: 0.769–0.953), significantly outperforming standalone models (Fig. 3A, D). DeLong testing confirmed statistically significant integrated discrimination improvement, the IDI ranged from 0.038 to 0.308 (all *p* < 0.05; Table 2). Across standard metrics, the combined model achieved optimal results in training (ACC = 0.723, SEN = 0.856, SPE = 0.778, PPV = 0.877, NPV = 0.689) and validation (ACC = 0.617, SEN = 0.867, SPE = 0.734, PPV = 0.840, NPV = 0.667) cohorts (Fig. 3M, N). DCA revealed enhanced clinical net benefit for the combined model across probability thresholds (Fig. 3B-C, E-F), while calibration curves demonstrated excellent agreement between predicted and observed recurrence probabilities across all groups. The predictive performance for recurrence outcomes was evaluated using confusion matrices, with the integrated model demonstrating superior classification accuracy (Fig. 3G-L).

In 100 repeated stratified 8:2 random splits, the delta-radiomics and combined models showed stable validation performance, with mean AUCs of 0.806 ± 0.052 and 0.842 ± 0.047 (Table S10), respectively. The primary-split AUCs fell within the repeated-split distributions, suggesting that model performance was not driven by a favorable random partition.

**Table 2:** The comparison of the performance of different prediction models using Delong test in the training and validation cohorts

Cohort	Model comparison	IDI (integrated discrimination improvement)	*p value
Training cohort	Radiomics Model vs. Clinical Model	0.209 (0.140–0.277)	< 0.001
	Combined Model vs. Clinical Model	0.250 (0.192–0.307)	< 0.001
	Combined Model vs. Radiomics Model	0.038 (0.012–0.064)	0.004
Validation cohort	Radiomics Model vs. Clinical Model	0.224 (0.084–0.365)	0.002
	Combined Model vs. Clinical Model	0.308 (0.189–0.426)	< 0.001
	Combined Model vs. Radiomics Model	0.083 (0.017–0.150)	0.014

*p value was calculated with the DeLong test

### Prognostic risk stratification

**Fig. 4:**
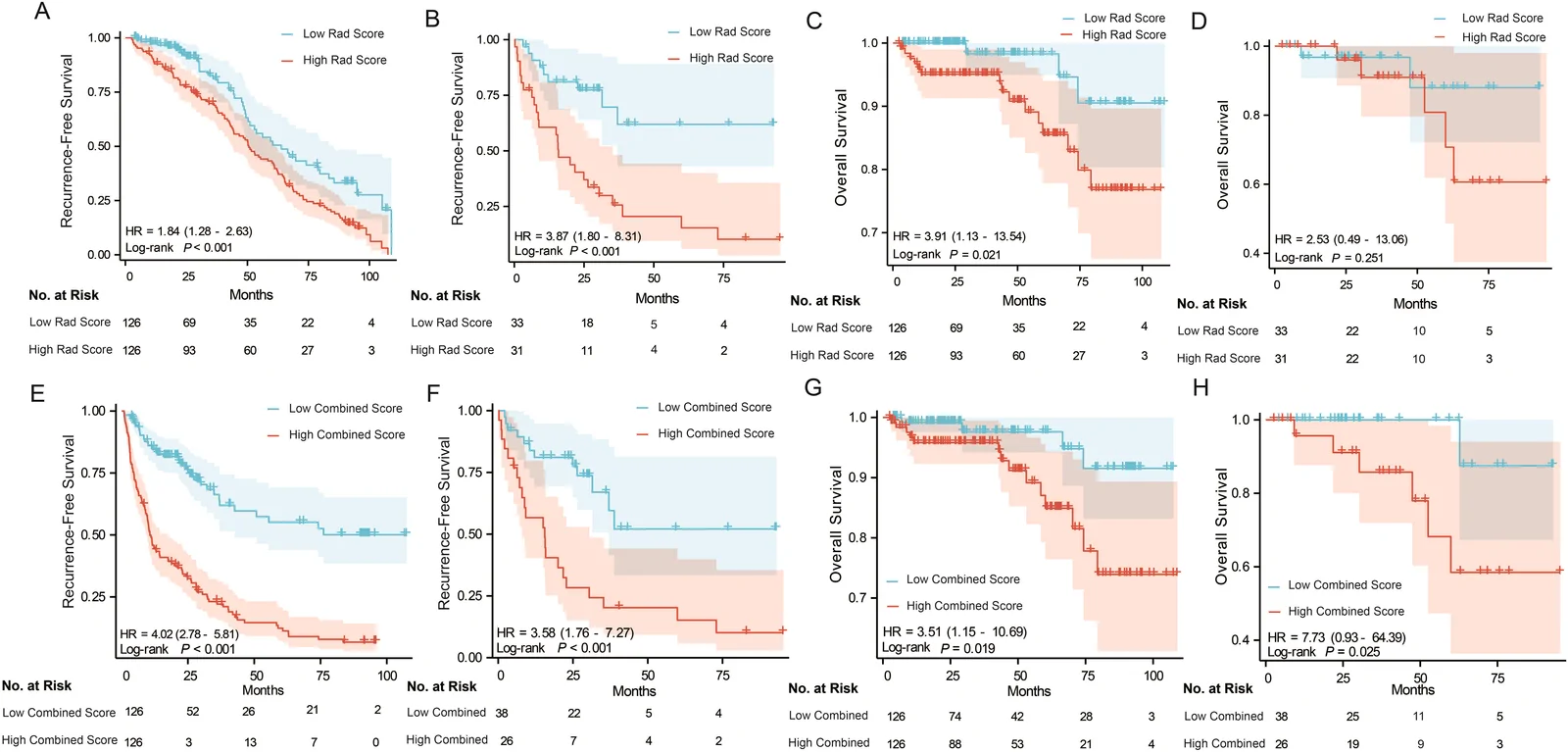
Kaplan-Meier curves in the outcome cohort. Delta radiomics model (**A**-**D**: RFS in training/validation cohorts **A**/**B**; OS in **C**/**D**) and combined model (**E**-**H**: RFS in training/validation **E**/**F**; OS in **G**/**H**). A high model score is represented by a red line and a low model score is represented by a blue line. RFS, recurrence-free survival. OS, overall survival

Successful stratification into high- and low-radscore groups was achieved using median thresholds for both delta-radiomics and combined models. In the delta-radiomics model, high-RadScore patients demonstrated significantly shorter RFS in the training cohort [median RFS: 50.56 vs. 63.88 months; HR = 1.84 (1.28–2.63), log-rank *p* < 0.001; Fig. 4A] and validation cohort [HR = 3.87 (1.80–8.31), log-rank *p* < 0.001; Fig. 4B], high-RadScore patients demonstrated significantly shorter OS in the training cohort[HR = 3.91 (1.13–13.54), log-rank *p* = 0.021; Fig. 4C] and validation cohort [HR = 2.53 (0.49–13.06), log-rank *p* = 0.251; Fig. 4D], though median OS remained unreachable in both groups (Fig. 4C, D).

Similarly, the combined model showed pronounced prognostic stratification: high-RadScore patients exhibited substantially shorter RFS in the training [median: 10.65 months; HR = 4.02 (2.78–5.81), log-rank *p* < 0.001; Fig. 4E] and validation cohorts [median: 15.55 months; HR = 3.58 (1.76–7.27), log-rank *p* < 0.001; Fig. 4F]. High-RadScore patients had shorter OS compared with low-RadScore patients, with median OS not reached in either group. In the training cohort, high-RadScore patients had HR = 3.51 (95% CI: 1.15–10.69, log-rank *p* = 0.019; Fig. 4G), and in the validation cohort, HR = 7.73 (95% CI: 0.93–64.39, log-rank *p* = 0.025; Fig. 4H). Detailed event counts and total patient numbers for each risk group are provided in Table S11.

### Finding functional genes for radiomics models

**Fig. 5:**
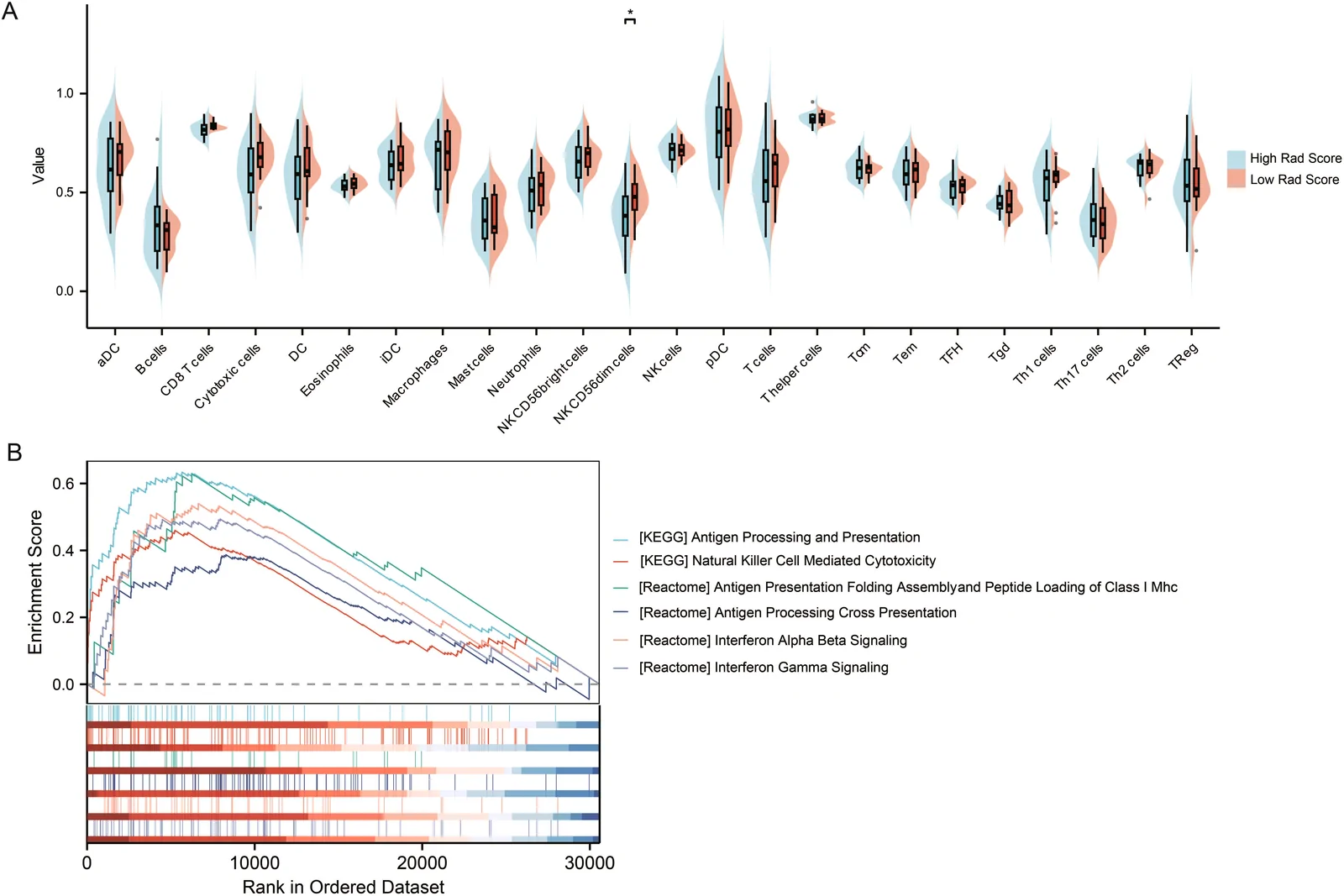
(**A**) Differences in immune infiltration between high and low Rad Score in the TCGA classification system. (**B**) Gene set enrichment analysis revealed significant oncogenic pathways associated with immune infiltration

The TCIA cohort comprised 33 bladder cancer patients with qualifying CT images, stratified by radiomics score using the median threshold established in the Union Hospital training set. Differential gene expression analysis identified and enriched recurrence-associated genes for subsequent investigation. Single-sample Gene Set Enrichment Analysis (ssGSEA) revealed distinct immune infiltration patterns, with significantly elevated NK CD56dim cell proportions in the low-RadScore group versus high-RadScore (Fig. 5A). Functional enrichment analysis demonstrated marked upregulation of key pathways in low-RadScore patients, including: natural killer cell mediated cytotoxicity, antigen presentation-folding assembly and peptide loading of class I MHC, antigen processing-cross presentation, interferon alpha/beta signaling, and interferon gamma signaling (Fig. 5B).

## Discussion

Despite radical cystectomy, bladder cancer exhibits substantial recurrence rates, particularly in muscle-invasive disease [25]. Current diagnostic and surveillance paradigms rely heavily on cystoscopy and cytology, which carry inherent limitations. This study successfully developed and validated a novel delta-radiomics approach leveraging pre-operative unenhanced and contrast-enhanced CT imaging, integrated with machine learning to predict post-surgical recurrence and enable risk stratification. Our principal findings demonstrate significant advances: (1) Delta-radiomic features capturing multiphasic tumor changes demonstrated superior predictive efficacy for recurrence (AUC range: 0.789–0.886); (2) Radiomic models implementing LightGBM, particularly when fused with clinical parameters, outperformed alternative models and isolated clinical factors; (3) Radscore-based stratification identified patient cohorts with significantly divergent RFS and OS; (4) Additionally, exploratory transcriptomic profiling showed enrichment of immune-related pathways in low-risk radiomic subgroups, providing preliminary biological context for their favorable prognosis. The presence or absence of recurrence-associated imaging signatures may reflect immunophenotypes capable of tumor infiltration. To our knowledge, this represents the first integrative study employing delta-radiomic features for bladder cancer recurrence prediction.

Radiomics provides a non-invasive approach to characterize tumor biology through high-throughput quantitative feature extraction from medical images. Recent studies increasingly employ CT- or MRI-based radiomic models for predicting OS and progression-free survival in bladder cancer. Zhang et al. [26] developed DWI-derived radiomic signatures predicting progression-free survival (PFS) in MIBC patients, achieving a C-index of 0.612 in external validation. Another investigation integrated radiomic features from tumor and nodal regions with clinical parameters to predict OS after radical cystectomy, reporting an average AUC of 0.785 over 1–7 years [27]. Radiological features based on enhanced CT imaging have also been shown to have significant potential in predicting prognosis in patients with muscle-invasive bladder cancer, with an external test group AUC of 0.730,95% CI (0.569–0.891) [28]. Our study demonstrates that single-phase radiomic models effectively predict bladder cancer recurrence, with validation cohort AUCs of 0.723 (95% CI: 0.594–0.851) for unenhanced phase features and 0.783 (95% CI: 0.666-0.900) for arterial phase features. Nevertheless, the conventional single-phase radiomics methodology applied in these studies remains limited to preselected morphological/textural characteristics, which may omit clinically valuable imaging data.

This study is among the first to apply CT-based delta-radiomics to postoperative recurrence prediction in bladder cancer. Previous studies have shown that delta-radiomics can provide additional predictive information beyond conventional single-phase radiomics by capturing dynamic changes in texture features across temporal, anatomical, or imaging-phase dimensions [19, 29, 30]. In this study, delta-radiomics features were calculated as RF_delta = RF_arterial − RF_unenhanced. Physiologically, unenhanced CT mainly reflects baseline tumor morphology, intrinsic attenuation, necrosis, hemorrhage, and anatomical background, whereas arterial-phase CT provides enhancement-related information associated with vascularity, perfusion, vascular permeability, and viable tumor heterogeneity. Mathematically, this subtraction may partly reduce baseline anatomical and density-related signals shared by both phases and highlight enhancement-associated changes in intensity distribution and texture [31]. These phase-dependent texture changes may better capture intratumoral heterogeneity, including heterogeneous vascular supply, necrotic or hypoxic regions, and uneven enhancement patterns.

Recent studies have also demonstrated the value of delta-radiomics in predicting treatment response in muscle-invasive bladder cancer [32] and prognosis in small cell lung cancer [33] and hepatocellular carcinoma [34]. Enhancement patterns may serve as imaging surrogates of tumor vascularity and microvessel density, which have been associated with recurrence and survival outcomes [35–37]. In our study, the delta-radiomics model showed stable discrimination, and the combined model integrating Rad-score with muscle invasion achieved AUCs of 0.860 and 0.861 in the training and validation cohorts, respectively. Nevertheless, delta-radiomics should be interpreted as an imaging-derived surrogate rather than a direct biological measurement, and further histopathological or multi-omics validation is needed.

Radiomics risk stratification separated patients into high- and low-RadScore groups. For the delta-radiomics model, high RadScore was associated with shorter RFS in both training (HR = 1.84, 95% CI: 1.28–2.63; *p* < 0.001) and validation cohorts (HR = 3.87, 95% CI: 1.80–8.31; *p* < 0.001), indicating consistent risk direction. OS differences were significant in training (HR = 3.91, *p* = 0.021) but not in validation (HR = 2.53, *p* = 0.251), likely due to fewer events and smaller sample size (*n* = 64; 33 low-/31 high-RadScore; see Table S11). In such modest cohorts, small differences in event counts or timing can inflate Cox HR estimates, as reflected in the wider CI for validation RFS. Overall, the delta-radiomics model confirms its ability to stratify patients for both RFS and OS, highlighting its potential clinical relevance in postoperative management. High-risk identification may guide intensified surveillance, whereas low-risk patients could avoid unnecessary interventions.

This study also explored whether CT-derived radiomic signatures were associated with tumor molecular biology, particularly the tumor immune microenvironment (TIME). In the TCIA cohort, tumors with low RadScore showed enrichment of immune-related pathways, including antigen processing and presentation, natural killer cell-mediated cytotoxicity, and interferon signaling involving IFN-alpha/beta and IFN-gamma pathways. These immune-active transcriptional programs may provide a biological context for the favorable prognosis observed in the low-radiomic-risk subgroup, as enhanced antigen presentation and NK cell activity are closely related to antitumor immune surveillance, whereas immune evasion is a recognized hallmark of cancer [38]. Thus, the observed radiomic risk pattern may partly reflect differences in tumor immune activity, providing preliminary biological support for the prognostic value of the delta-radiomics model.

Several limitations should be acknowledged. First, the study’s retrospective single-center design may introduce selection bias, and prospective multicenter validation is needed to confirm generalizability. Although interobserver analysis showed good segmentation agreement, this assessment was limited to the current cohort. Moreover, because the imaging data were acquired at a single institution with broadly consistent CT protocols, formal inter-center batch-effect correction was not performed; residual scanner- or protocol-related variability may still affect radiomic feature stability. Future multicenter studies should employ harmonized imaging protocols and radiomics harmonization techniques to validate model robustness. Second, the internal validation cohort was small (*n* = 64; 33 low- and 31 high-RadScore patients), and the modest subgroup sizes with imbalanced event counts or timing may yield unstable HR estimates, partly explaining the relatively high validation RFS HR. Third, only CT imaging was used; integrating multimodal data, such as MRI or histopathology, could further improve predictive performance. Finally, the transcriptomic analysis was limited by the small external TCIA cohort (*n* = 33) and the absence of paired pathological immune profiling. Therefore, the observed immune-related pathway enrichment in the low-RadScore group should be considered exploratory, and further validation in larger cohorts with paired imaging, transcriptomic, and pathological or immunohistochemical data is warranted.

## Conclusion

Delta-radiomics analysis of pre-operative unenhanced and contrast-enhanced CT scans enabled development of predictive models for bladder cancer recurrence assessment. This approach demonstrated superior performance compared to conventional single-phase radiomics, while the integrated clinico-radiomic model achieved optimal diagnostic efficacy for pre-surgical recurrence prediction. This combined methodology represents a promising imaging biomarker strategy to guide personalized therapeutic decision-making.

## Supplementary Information

Below is the link to the electronic supplementary material.

Supplementary Material 1

## Data Availability

The datasets used and/or analyzed during the current study are available from the corresponding author [Lian Yang, e-mail yanglian@hust.edu.cn] upon reasonable request.

## Abbreviations

ACC: Accuracy
AGR: Calbumin to globulin ratio
AUC-ROC: Area under the receiver operating characteristic curve
BLCA: Bladder cancer
BMI: Body mass index
CT: Computed tomography
CR: Complete remission
DCA: Decision curve analysis
delta-RFs: Delta-radiomics features
ET: Extra Trees
IBSI: Image Biomarker Standardization Initiative
ICC: Intraclass correlation coefficient
IDI: Integrated discrimination improvement
Inte-prob: Integrated probability
KNN: k-Nearest Neighbors
LASSO: Least absolute shrinkage and selection operator
LightGBM: Light Gradient Boosting Machine
LR: Logistic Regression
MIBC: Muscle-invasive bladder cancer
MLP: Multilayer Perceptron
NLR: Neutrophil-to-lymphocyte ratio
NMIBC: Non-muscle-invasive bladder cancer
NPV: Negative predictive value
OS: Overall survival
PLR: Platelet-to-lymphocyte ratio
PPV: Positive predictive value
Rad-prob: Radiomics-derived probability
RF: Random Forest
RFS: Recurrence-free survival
ROI: Regions of interest
SEN: Sensitivity
SPE: Specificity
ssGSEA: Single-sample Gene Set Enrichment Analysis
SVM: Support Vector Machine
TCIA: The Cancer Image Archive
TURBT: Transurethral resection of bladder tumours
VOI: Volume of interest
