# Supplementary material for: Development of multiphasic CT-based delta-radiomics model for predicting postoperative recurrence risk in bladder cancer
Source: BMC Med Imaging. 2026 Jul 7;26:478. doi: 10.1186/s12880-026-02554-2 (PMC13621731; doi:10.1186/s12880-026-02554-2)
Supplement: Supplementary file 1 [file 12880_2026_2554_MOESM1_ESM.docx]

**Supplementary Materials of Figures and Table**

**Supplementary Materials Tables**

Table S1. CT scanning protocol for bladder cancer.

Table S2. Characteristics cohort of patients in the TCIA group.

Table S3. Interobserver agreement of AI-assisted manually refined tumor VOIs assessed using Dice coefficient and ICC.

Table S4. LASSO-selected delta-radiomics features and coefficients used for Rad-score construction.

Table S5. Hyperparameters of the eight machine-learning classifiers used for delta-radiomics model development.

Table S6. The performance of delta radiomics models constructed by nine ML classifiers in postoperative patients with BLCA in the training and validation cohorts.

The performance of delta radiomics models constructed by nine ML classifiers in postoperative patients with BLCA in the training and validation cohorts.

Table S7. The comparison of the performance of different prediction models using Delong test in the training and validation cohorts.

Table S8. Univariate and multivariate logistic regression analysis of RFS.

Table S9. Final selected hyperparameters of the seven machine-learning classifiers evaluated for the combined clinico-radiomic model.

Table S10. Performance Stability of the Delta-Radiomics and Combined Clinical-Radiomics Models Across 100 Repeated Stratified 8:2 Random Splits.

Table S11. Event distribution and Kaplan-Meier survival stratification according to risk groups in the Delta-radiomics and combined models.

**Supplementary Materials Figures**

Figure S1. Analysis of imaging phases.

Figure S2. Feature importance analysis of the delta radiomics model.

Figure S3. Comparative Performance Assessment of three Radiomics Models.

Figure S4. Pertormance comparison of machine-eaming algorithms for the combined clinico-radiomic model.

Figure S5. Performance and comparison of prediction models in the training and validation cohorts.

**Table S1.** **CT scanning protocol for bladder cancer.**

| Parameter | Unenhanced CT | Arterial phase | Venous phase | Equilibrium phase |
| --- | --- | --- | --- | --- |
| Basic settings |  |  |  |  |
| Collimation (mm) | 64 x 0.625 / 32 x 1.2 / 48 x 1.2 | 64 x 0.625 / 32 x 1.2 / 48 x 1.2 | 64 x 0.625 / 32 x 1.2 / 48 x 1.2 | 64 x 0.625 / 32 x 1.2 / 48 x 1.2 |
| Rotation time (s) | 0.75/0.5 | 0.75/0.5 | 0.75/0.5 | 0.75/0.5 |
| Helical pitch | 0.891/0.8 | 0.891/0.8 | 0.891/0.8 | 0.891/0.8 |
| Slice thickness(mm) | 1.5/1.2 | 1.5/1.2 | 1.5/1.2 | 1.5/1.2 |
| Contrast-enhanced scanning | | | | |
| Dosage | | 300~350mg/ml | | |
| Rate (mL/s) |  | 2~3ml/s | | |
| Acquisition time (s)* |  | 30~35s | 60~80s |  |
| Coverage | Scanning from the superior pole of the kidney to the inferior border of the pubic symphysis | | | |

*Acquisition time means time interval from contrast injection to image acquisition

**Table S2. Characteristics cohort of patients in the TCIA group.**

| Patient characteristics (n=33) | Number of patients (%) | |
| --- | --- | --- |
| Patient characteristics |  |  |
| Gender, n (%) |  |  |
| Male | 24 | 72.7% |
| Female | 9 | 27.3% |
| Pathologic tumor grade,n (%) |  |  |
| Low grade/ PUNLMP | 0 | 0% |
| High grade | 33 | 100% |
| Pathologic T stage, n (%) |  |  |
| < T2 | 0 | 0% |
| ≥ T2 | 30 | 100% |
| Age, mean (SD) | 69.8 | 10.7 |
| Smoking, n (%) |  |  |
| Current smoker | 3 | 9.4% |
| Never smoker | 7 | 21.9% |
| Former smoker | 22 | 68.8% |

Abbreviations: SD, standard deviation; PUNLMP, papillary urothelial neoplasms of low malignant potential.

**Table S3. Interobserver agreement of AI-assisted manually refined tumor VOIs assessed using Dice coefficient and ICC.**

| Metric | Segmentation Performance | 95% Confidence Interval | SD |
| --- | --- | --- | --- |
| Dice Coefficient | 0.88 | 0.86-0.90 | 0.04 |
| ICC for Segmentation-Derived Measurements | 0.94 | 0.93-0.95 | N/A |

Abbreviations: ICC, intraclass correlation coefficient; SD, standard deviation.

**Table S4. LASSO-selected delta-radiomics features and coefficients used for Rad-score construction.**

| No. | Feature name | Filter / transformation | Feature category | Feature descriptor | LASSO coefficient |
| --- | --- | --- | --- | --- | --- |
| 1 | lbp_3D_m2_firstorder_Skewness_Delta | LBP-3D-m2 | First-order | Skewness | 0.022886 |
| 2 | log_sigma_1_0_mm_3D_firstorder_10Percentile_Delta | LoG, sigma = 1.0 mm, 3D | First-order | 10th percentile | 0.019835 |
| 3 | log_sigma_2_0_mm_3D_ngtdm_Strength_Delta | LoG, sigma = 2.0 mm, 3D | Texture / NGTDM | Strength | -0.036864 |
| 4 | original_shape_Maximum2DDiameterSlice_Delta | Original image | Shape | Maximum 2D diameter, slice | 0.004382 |
| 5 | squareroot_ngtdm_Strength_Delta | Square-root transformed image | Texture / NGTDM | Strength | -0.015045 |
| 6 | wavelet_HHH_firstorder_InterquartileRange_Delta | Wavelet-HHH | First-order | Interquartile range | -0.019155 |
| 7 | wavelet_HLL_firstorder_Kurtosis_Delta | Wavelet-HLL | First-order | Kurtosis | 0.030963 |
| 8 | wavelet_LLH_firstorder_Skewness_Delta | Wavelet-LLH | First-order | Skewness | -0.045098 |
| 9 | wavelet_LLH_ngtdm_Strength_Delta | Wavelet-LLH | Texture / NGTDM | Strength | -0.010462 |

**Table S5. Hyperparameters of the eight machine-learning classifiers used for delta-radiomics model development.**

| No. | Model name in code | Classifier | Explicit hyperparameters in the code |
| --- | --- | --- | --- |
| 1 | LR | LogisticRegression | penalty='none'; max_iter=100 |
| 2 | SVM | SVC | probability=True; max_iter=100 |
| 3 | RandomForest | RandomForestClassifier | n_estimators=50; max_depth=5; min_samples_split=4; random_state=0 |
| 4 | XGBoost | XGBClassifier | n_estimators=100; objective='binary:logistic'; max_depth=3; min_child_weight=0.2; use_label_encoder=False; eval_metric='error' |
| 5 | LightGBM | LGBMClassifier | n_estimators=20; max_depth=4; min_child_weight=0.5 |
| 6 | ExtraTrees | ExtraTreesClassifier | n_estimators=20; max_depth=5; min_samples_split=2; random_state=0 |
| 7 | KNN | KNeighborsClassifier | n_neighbors=5; weights='uniform';  metric='minkowski'; p=2 |
| 8 | MLP | MLPClassifier | hidden_layer_sizes=(100,); activation='relu'; solver='adam'; alpha=0.0001; learning_rate_init=0.001; max_iter=500; random_state=0 |

**Table S6. The performance of delta radiomics models constructed by eight ML classifiers in postoperative patients with BLCA in the training and validation cohorts.**

| Model  name | Cohort | ACC | AUC | 95%CI | SEN | SPE | PPV | NPV | Precision | Recall | F1 score |
| --- | --- | --- | --- | --- | --- | --- | --- | --- | --- | --- | --- |
| LR | Training | 0.615 | 0.635 | 0.564-0.706 | 0.628 | 0.596 | 0.689 | 0.530 | 0.689 | 0.628 | 0.657 |
| LR | Validation | 0.750 | 0.788 | 0.673-0.904 | 0.676 | 0.833 | 0.821 | 0.694 | 0.821 | 0.676 | 0.742 |
| SVM | Training | 0.762 | 0.802 | 0.743-0.860 | 0.764 | 0.760 | 0.819 | 0.693 | 0.819 | 0.764 | 0.790 |
| SVM | Validation | 0.672 | 0.688 | 0.556-0.820 | 0.471 | 0.900 | 0.842 | 0.600 | 0.842 | 0.471 | 0.604 |
| KNN | Training | 0.496 | 0.769 | 0.715-0.824 | 0.142 | 1.000 | 1.000 | 0.450 | 1.000 | 0.142 | 0.249 |
| KNN | Validation | 0.469 | 0.491 | 0.352-0.630 | 0.000 | 1.000 | 0.000 | 0.469 | 0.000 | 0.000 | NaN |
| RandomForest | Training | 0.758 | 0.796 | 0.741-0.852 | 0.865 | 0.606 | 0.757 | 0.759 | 0.757 | 0.865 | 0.808 |
| RandomForest | Validation | 0.656 | 0.706 | 0.577-0.835 | 0.441 | 0.900 | 0.833 | 0587 | 0.833 | 0.441 | 0.577 |
| ExtraTrees | Training | 0.571 | 0.657 | 0.591-0.723 | 0.480 | 0.702 | 0.696 | 0.487 | 0.696 | 0.480 | 0.568 |
| ExtraTrees | Validation | 0.516 | 0.590 | 0.453-0.726 | 0.147 | 0.933 | 0.714 | 0.491 | 0.714 | 0.147 | 0.244 |
| XGBoost | Training | 0.861 | 0.928 | 0.897-0.958 | 0.892 | 0.817 | 0.874 | 0.842 | 0.874 | 0.892 | 0.883 |
| XGBoost | Validation | 0.703 | 0.753 | 0.632-0.874 | 0.824 | 0.567 | 0.683 | 0.739 | 0.683 | 0.824 | 0.747 |
| LightGBM | Training | 0.738 | 0.837 | 0.789-0.886 | 0.669 | 0.837 | 0.853 | 0.640 | 0.853 | 0.669 | 0.750 |
| LightGBM | Validation | 0.750 | 0.822 | 0.717-0.926 | 0.706 | 0.800 | 0.800 | 0.706 | 0.800 | 0.706 | 0.750 |
| MLP | Training | 0.655 | 0.720 | 0.656-0.783 | 0.561 | 0.788 | 0.790 | 0.558 | 0.790 | 0.561 | 0.656 |
| MLP | Validation | 0.812 | 0.814 | 0.705-0.923 | 0.853 | 0.767 | 0.806 | 0.821 | 0.806 | 0.853 | 0.829 |

Abbreviations: ML, machine learning; LR, logistic regression; SVM, support vector machine; KNN, k-nearest neighbor; RF, random forest; LightGBM, light gradient boosting machine; XGBoost, eXtreme Gradient Boosting; MLP, multilayer perceptron; ACC, accuracy; AUC, area under the receiver operating curve; SEN, sensitivity; SPE, specificity; PPV, positive predictive value; NPV, negative predictive value; CI, confidence interval.

**Table S7. The comparison of the performance of different prediction models using Delong test in the training and validation cohorts.**

| **Cohort** | **Model comparison** | **IDI (integrated discrimination improvement)** | ****p* value** |
| --- | --- | --- | --- |
| Training cohort | LightGBM vs. RandomForest | 0.032 (0.011-0.054) | 0.092 |
|  | LightGBM vs. XGBoost | 0.149 (0.125-0.174) | <0.001 |
|  | LightGBM vs. MLP Model | -0.020 (-0.400-0.000) | <0.001 |
| Validation cohort | LightGBM vs. RandomForest | 0.000 (-0.045-0.049) | 0.030 |
|  | LightGBM vs. XGBoost | 0.046 (-0.005-0.094) | 0.18 |
|  | LightGBM vs. MLP Model | 0.011 (-0.028-0.053) | 0.89 |

****p* value was calculated with the DeLong test.**

**Table S8. Univariate and multivariate logistic regression analysis of RFS.**

| Parameter | Univariate analysis | | Multivariate analysis | |
| --- | --- | --- | --- | --- |
|  | Odds Ratio (95% CI) | *P* value | Odds Ratio (95% CI) | *P* value |
| Gender |  |  |  |  |
| Male | Reference |  |  |  |
| Female | 0.991 (0.502 - 1.953) | 0.978 |  |  |
| Age | 1.006 (0.987 - 1.026) | 0.532 |  |  |
| Pathologic T stage |  |  |  |  |
| < T2 | Reference |  | Reference |  |
| ≥ T2 | 7.885 (3.693 - 16.837) | < 0.001 | 7.151 (3.072 - 16.649) | < 0.001 |
| Pathologic tumor grade |  |  |  |  |
| Low grade/ PUNLMP | Reference |  | Reference |  |
| High grade | 2.348 (1.405 - 3.925) | 0.001 | 0.975 (0.529 - 1.795) | 0.935 |
| Adjuvant chemotherapy |  |  |  |  |
| No | Reference |  | Reference |  |
| Yes | 0.518 (0.307 - 0.875) | 0.014 | 0.605 (0.339 - 1.080) | 0.089 |
| Body mass index | 0.980 (0.902 - 1.064) | 0.632 |  |  |
| Cardiovascular disease |  |  |  |  |
| No | Reference |  |  |  |
| Yes | 0.746 (0.316 - 1.762) | 0.504 |  |  |
| Hypertension |  |  |  |  |
| No | Reference |  |  |  |
| Yes | 0.843 (0.483 - 1.471) | 0.547 |  |  |
| Diabetes |  |  |  |  |
| No | Reference |  |  |  |
| Yes | 0.662 (0.326 - 1.343) | 0.253 |  |  |
| Drinking |  |  |  |  |
| No | Reference |  |  |  |
| Yes | 1.831 (0.970 - 3.457) | 0.062 |  |  |
| Smoking |  |  |  |  |
| No | Reference |  |  |  |
| Yes | 1.238 (0.722 - 2.124) | 0.438 |  |  |
| NLR | 1.089 (0.990 - 1.197) | 0.079 |  |  |
| PLR | 1.004 (1.001 - 1.008) | 0.026 | 1.001 (0.996 - 1.007) | 0.600 |
| AGR | 0.918 (0.433 - 1.947) | 0.823 |  |  |

Abbreviations: NLR, neutrophil-to-lymphocyte ratio; PLR, platelet-to-lymphocyte ratio; A/G ratio, albumin to globulin ratio; BMI, body mass index; PUNLMP, Papillary Urothelial Neoplasms of Low Malignant Potential.

**Table S9. Final selected hyperparameters of the seven machine-learning classifiers evaluated for the combined clinico-radiomic model.**

| Model | Best parameters |
| --- | --- |
| Logistic regression | {} |
| Ridge logistic regression | {"model__C": 0.1} |
| Elastic-net logistic regression | {"model__C": 0.1, "model__l1_ratio": 0.7} |
| Linear SVM | {"model__C": 1} |
| Random Forest | {"model__max_depth": 3, "model__max_features": "sqrt", "model__min_samples_leaf": 20, "model__min_samples_split": 10, "model__n_estimators": 500} |
| XGBoost | {"model__subsample": 0.8, "model__reg_lambda": 1, "model__reg_alpha": 1, "model__n_estimators": 50, "model__min_child_weight": 10, "model__max_depth": 2, "model__learning_rate": 0.05, "model__colsample_bytree": 0.8} |
| LightGBM | {"model__subsample": 1.0, "model__reg_lambda": 5, "model__reg_alpha": 0.1, "model__num_leaves": 5, "model__n_estimators": 100, "model__min_child_samples": 10, "model__max_depth": 1, "model__learning_rate": 0.1, "model__colsample_bytree": 0.8} |

**Table S10. Performance Stability of the Delta-Radiomics and Combined Clinical-Radiomics Models Across 100 Repeated Stratified 8:2 Random Splits.**

| Model | Mean validation AUC ± SD | Median AUC | IQR | 95%CI | Primary split AUC |
| --- | --- | --- | --- | --- | --- |
| Delta-radiomics model | 0.806 ± 0.052 | 0.812 | 0.774–0.846 | 0.701–0.895 | 0.822 |
| Combined clinical-radiomics model | 0.842 ± 0.047 | 0.845 | 0.812–0.875 | 0.742–0.921 | 0.861 |

**Table S11. Event distribution and Kaplan-Meier survival stratification according to risk groups in the Delta-radiomics and combined models.**

| Model | Cohort | Outcome | Risk group | Events / total patients | HR (95% CI) | Log-rank P value |
| --- | --- | --- | --- | --- | --- | --- |
| Delta-radiomics | Training | RFS | Low Rad-score | 44 / 126 | Reference | — |
| Delta-radiomics | Training | RFS | High Rad-score | 104 / 126 | 1.84 (1.28–2.63) | <0.001 |
| Delta-radiomics | Validation | RFS | Low Rad-score | 9 / 33 | Reference | — |
| Delta-radiomics | Validation | RFS | High Rad-score | 25 / 31 | 3.87 (1.80–8.31) | <0.001 |
| Delta-radiomics | Training | OS | Low Rad-score | 3 / 126 | Reference | — |
| Delta-radiomics | Training | OS | High Rad-score | 15 / 126 | 3.91 (1.13–13.54) | 0.021 |
| Delta-radiomics | Validation | OS | Low Rad-score | 2 / 33 | Reference | — |
| Delta-radiomics | Validation | OS | High Rad-score | 5 / 31 | 2.53 (0.49–13.06) | 0.251 |
| Combined | Training | RFS | Low Combined score | 39 / 126 | Reference | — |
| Combined | Training | RFS | High Combined score | 109 / 126 | 4.02 (2.78–5.81) | <0.001 |
| Combined | Validation | RFS | Low Combined score | 18 / 38 | Reference | — |
| Combined | Validation | RFS | High Combined score | 22 / 26 | 3.58 (1.76–7.27) | <0.001 |
| Combined | Training | OS | Low Combined score | 4 / 126 | Reference | — |
| Combined | Training | OS | High Combined score | 14 / 126 | 3.51 (1.15–10.69) | 0.019 |
| Combined | Validation | OS | Low Combined score | 1 / 38 | Reference | — |
| Combined | Validation | OS | High Combined score | 6 / 26 | 7.73 (0.93–64.39) | 0.025 |

Abbreviations: RFS, recurrence-free survival; OS, overall survival; HR, hazard ratio; CI, confidence interval.
